# Paradoxical CD4 Lymphopenia in Autoimmune Lymphoproliferative Syndrome (ALPS)

**DOI:** 10.3389/fimmu.2019.01193

**Published:** 2019-05-29

**Authors:** Andrea Lisco, Chun-Shu Wong, Susan Price, Peiying Ye, Julie Niemela, Megan Anderson, Elizabeth Richards, Maura Manion, Harry Mystakelis, Morgan Similuk, Bernice Lo, Jennifer Stoddard, Sergio Rosenzweig, Christophe Vanpouille, Adam Rupert, Irina Maric, Ainhoa Perez-Diez, David Parenti, Peter D. Burbelo, V. Koneti Rao, Irini Sereti

**Affiliations:** ^1^Laboratory of Immunoregulation, National Institute of Allergy and Infectious Diseases (NIAID), National Institutes of Health (NIH), Bethesda, MD, United States; ^2^Laboratory of Clinical Immunology and Microbiology, National Institute of Allergy and Infectious Diseases, National Institutes of Health, Bethesda, MD, United States; ^3^Immunology Service, Department of Laboratory Medicine, National Institutes of Health, Bethesda, MD, United States; ^4^Clinical Genomics Program, National Institute of Allergy and Infectious Diseases, National Institutes of Health, Bethesda, MD, United States; ^5^Laboratory of Immunology, National Institute of Allergy and Infectious Diseases, National Institutes of Health, Bethesda, MD, United States; ^6^Program in Physical Biology, Eunice Kennedy-Shriver National Institute of Child Health and Human Development, National Institutes of Health, Bethesda, MD, United States; ^7^AIDS Monitoring Laboratory, Leidos Biomedical Research, Frederick, MD, United States; ^8^Hematology Service, Department of Laboratory Medicine, National Institutes of Health, Bethesda, MD, United States; ^9^George Washington University Medical Center, Washington, DC, United States; ^10^Dental Clinical Research Core, National Institute of Dental and Craniofacial Research, National Institutes of Health, Bethesda, MD, United States

**Keywords:** CD4 lymphopenia, follicular T helper cells, ALPS-FAS, autoimmune cytopenia, apoptosis

## Abstract

Autoimmune lymphoproliferative syndrome (ALPS) is caused by germline or somatic loss of function *FAS* mutations resulting in impaired apoptosis and consequent expansion of T-lymphocytes causing organomegaly and autoimmune anemia, neutropenia and thrombocytopenia. Herein, we report on a case of disseminated varicella zoster infection after post-partum vaccination in a patient found to have CD4 lymphopenia and eventually diagnosed with ALPS caused by a novel germline missense mutation in *FAS* death-domain. A subsequent retrospective analysis of 169 patients of the NIH ALPS-FAS cohort, revealed that CD4-T-cells lymphopenia (< 300 cells/μl) may occur in 5% of ALPS-FAS patients irrespectively of the underlying genetic defect, organomegaly or immunosuppressive treatment. Although immunophenotyping did not show depletion of specific CD4-T-cells subpopulations, CD4-lymphopenic ALPS-FAS subjects had an expansion of a subset of circulating T-follicular-helper (cTfh) cells, associated with autoantibody production (CCR7^low^PD-1^high^). Furthermore, autoantibodies binding on CD4-T-cells were detected in 50% of the CD4-lymphopenic ALPS-FAS patients and caused cytotoxicity in a natural killer (NK)-mediated antibody-dependent-cellular cytotoxicity assay. Such autoantibodies can therefore be associated with CD4-T-cell death, impaired activation induced proliferation or impaired trafficking. The expansion of autoreactive T-cells in ALPS-FAS is known to be associated with autoimmune clinical manifestations, however our study reveals that ALPS-FAS can also be associated with a paradoxical depletion of CD4-T-cells due to the presence of autoantibodies on the surface of CD4-T-cells which can in turn result in increased susceptibility to opportunistic infections. These novel findings have implications for the diagnosis, clinical monitoring, and management of patients with ALPS-FAS.

## Introduction

The number and reactivity of lymphocytes is tightly regulated so that prompt and efficient adaptive immune responses can be mounted. The clinical implications of genetic defects in the molecular mechanisms involved in the development, expansion, and contraction of T cell populations are clearly evident in primary immune deficiency diseases (PIDDs) in which opportunistic infections and adverse events to attenuated vaccines often characterize the initial clinical presentation (1).

The autoimmune lymphoproliferative syndrome (ALPS) in its most common variant, is caused by germline loss of function mutations in the *FAS* gene (2, 3). The FAS protein is expressed as surface homotrimers on T cells, interacting with homotrimerized FAS ligand (FAS-L) and initiates a cascade of caspase cleavages resulting in programmed cell death or apoptosis. In ALPS-FAS, the impaired apoptosis and the consequent expansion of specific subsets of lymphocytes (4) (i.e., total CD3^+^ T-cells, CD3^+^CD8^+^ T-cells, CD3^+^TCRαβ^+^CD4^−^CD8^−^ double negative T-cells, CD5^+^CD20^+^ B-cells), including self-antigen reactive subpopulations, results in specific clinical manifestations and laboratory abnormalities such as lymphadenopathy, hepatomegaly, splenomegaly, autoimmune hemolytic anemia, thrombocytopenia, and neutropenia (5, 6). Therefore, ALPS is classified among the PIDD as a ‘disease of immune dysregulation' as its clinical presentation is not due to an immunological defect *per se*, but rather to the clinical and immunological consequences of the accumulation of T and B lymphocytes (7–9).

Herein, we report on an index patient referred to NIH for evaluation of idiopathic CD4 lymphopenia (ICL) (10), who was eventually diagnosed with ALPS and a case series of patients with confirmed ALPS-FAS and paradoxical CD4 lymphopenia. We found that ALPS-FAS patients with CD4 lymphopenia have autoantibodies against CD4-T-cells causing NK-mediated antibody-dependent-cellular-cytotoxicity (ADCC) and that the presence of such autoantibodies is associated with an expansion of the CCR7^low^PD-1^high^ circulating T-follicular-helper cells (cTfh) subset that have been most recently associated with autoantibody production (11).

The present work highlights the complexity of the homeostatic regulation of T-cells subpopulation and the apparent paradox between a lymphoproliferative disorder and CD4 lymphopenia, along with the heterogeneity of clinical and laboratory findings in ALPS-FAS with implications for its diagnosis and management. Furthermore, the identification of a novel mechanism causing severe depletion of CD4 T-cells may suggest a potential role of auto-antibodies in ICL, HIV-1 infection, and other autoimmune diseases associated to a not yet explained reduction of CD4 T cells [i.e., Sjogren syndrome or systemic lupus erythematous (SLE)] (12).

## Materials and Methods

### Study Population

All patients provided written informed consent prior to study entry under NIH Clinical Center institutional review board-approved protocols (https://clinicaltrials.gov/ct2/show/NCT00001350; https://clinicaltrials.gov/ct2/show/NCT00867269).

Demographics and clinical characteristics of the enrolled patients are presented in Table 1. The index patient and 7 additional subjects with ALPS-FAS and CD4 cell count < 300 cells/μl were identified among the 169 subjects enrolled in the NIH ALPS-FAS cohort and were included in the present study. For each case, 2 ALPS-FAS subject controls matched for age and type of underlying FAS genetic defect were enrolled.

**Table 1:** Demographic, clinical, immunological, and genetic characteristics of the enrolled patients.

**Patients**	**Age**	**CD4 cells/μL (%)**	**Splenomegaly**	FAS mutation type^*^	**NM_000043**
Pt 1 Index	34	**111 (24)**	Yes	DD	719 T->A; M240K
Control-1A	43	938 (50)	No	DD	700(-6) C->G; 742T->G
Control-1B	35	731 (37)	Asplenia	DD	763(-5) T->G
Pt 2	30	**299 (26)**	Yes	Extracellular D	467 C->A; Y75X
Control-2A	25	775 (33)	Yes	Extracellular D	498 A->G; R86G
Control-2B	27	790 (39)	Yes	Extracellular D	535 A->T; E98splice
Pt 3	44	**257 (28)**	Yes	DD	952 G->T; G237V
Control-3A	47	1543 (46)	Asplenia	DD	960 G->A; E240K
Control-3B	51	773 (44)	No	DD	972 G->A; D244N
Pt.4	8	**216 (20)**	Yes	DD Somatic DNT	846(-1) G->T splicing
Control-4A	8	2063 (23)	Asplenia	DD Somatic DNT	870 G->T, 201fs
Control-4B	8	836 (52)	Yes	DD Somatic DNT	846(-9) del11
Pt.5	7	**265 (29)**	Yes	TM domain	526 A->GH95R
Control-5A	13	579 (39)	Yes	TM domain	526 A->GH95R
Control-5B	10	762 (45)	Yes	TM domain	528 (+3) A->C
Pt.6	11	**254 (14)**	No	DD	700(-16) A->G
Control-6A	11	417 (30)	Yes	DD	700(-6) C->G; 742T->G
Control-6B	12	972 (34)	Yes	DD	700(-3) C->T
Pt.7	7	**294 (28)**	No	DD	943 G->A; R234Q
Control-7A	8	639 (36)	Yes	DD	942C->T; R234X
Control-7B	8	504 (42)	Yes	DD	916 C>A; T225K
Pt.8	9	**244 (18)**	Yes	DD	970 T->C; I243T
Control-8A	6	992 (46)	Yes	DD	928 T>G; V229G
Control-8B	8	369 (35)	Yes	DD	998del14; Q252fs

* *DD, mutation in FAS death domain; extracellular D, mutation in the extracellular domain; DD, somatic DNT, somatic mutation in the double negative CD3+ T cells subset; TM domain, mutation in the transmembrane domain. Bold indicates CD4 cell counts and percentages in patients with ALPS and CD4 lymphopenia*.

### Immunophenotyping

Immunophenotyping of cryopreserved PBMC with staining of extracellular and intracellular antigens was performed with monoclonal antibodies from BD Biosciences (San Jose, CA) as previously described. Sufficient cryopreserved PBMC were available from 7 of the 8 cases and cells were stained with anti-CD3-V500; anti-CD4-BV605; anti-CD8-PacificBlue; anti-CD95-PeCy5; anti-CD127-PeCy7; anti-CD25-APC; anti-CD45RO-ECD or anti-CD45RO-PeCy7; anti-CD27-BV711; anti-Ki67-FITC; anti-FoxP3-PE; anti-TCRαβ-PE; anti-TCRγδ-FITC; anti-CD31-PE; anti-CD57-ECD; anti-B220-BV570; anti-PD-1-BV605; anti-CCR7-FITC; anti-CXCR5-PerCPCy5.5; anti-CXCR3-PE; and analyzed on a Fortessa flow-cytometer (BD Biosciences). Flowjo software 10.4.2 was used to analyze the data. We identified and excluded dead cells from the analysis using the LIVE/DEAD fixable Blue Dead Cell Stain kit (Invitrogen) and identified lymphocytes according to their light-scattering properties.

### FAS-Induced Apoptosis

FAS-induced apoptosis was evaluated using a FAS crosslinking assays as previously described (13). Briefly, 300,000 PBMC per each experimental condition were stimulated with different concentrations of a mouse anti-human FAS monoclonal antibody (Clone Apo 1-3, IgG3 Enzo Life Sciences) in the presence of a rat anti-mouse anti-IgG3 monoclonal antibody (Santa Cruz Biotechnology). The expression of AnnexinV was used to identify apoptotic cells with AnnexinV-APC labeled monoclonal antibody (Invitrogen) used according to the manufacturer instructions. Cells were incubated for 4 h and then washed, permeabilized and stained in 2 different monoclonal antibody panels to detect apoptosis in terminal effector memory T cells (TEM) with anti-CD3 PerCP-Cy5.5; CD27-PE; CD4-PeCy7; CD45RA-eFluor450 CCR7-FITC; AnnexinV-APC; and in double negative CD3^+^CD4^−^CD8^−^ αβ T cells (DNT) with anti-CD3eFluor450; CD4-APCCy7; CD8-BV605; TCRαβ-FITC. The fold changes in the expression of AnnexinV compared to non-stimulated control was used to evaluate FAS-induced apoptosis at different concentration of anti-FAS crosslinking antibody.

### FAS-Induced Apoptosis and Activation-Induced Cell Death (AICD) in T Cell Blasts

T cell blasts were generated as previously described and used in a FAS crosslinking and AICD assays (14–16). Briefly, PBMC were stimulated with Phytohaemagglutinin (PHA, 50 μg/ml, Millipore-Sigma) in complete RPMI with 10% human type AB serum. After wash-out of the mitogen, PBMC were resuspended in complete medium supplemented with 30 U/mL of recombinant human IL-2 (Millipore-Sigma). Fresh medium was added every 48 h and T cell blasts were used for apoptosis and AICD assays at day 3 of culture: For Fas-induced apoptosis, a mouse anti-human FAS monoclonal antibody (Clone CH11; Millipore-Sigma), was added for 16 h at different concentrations (0, 50, 100, 300, and 1,000 ng/mL) while sensitivity to AICD was evaluated by re-incubating T cell blasts with different concentrations of PHA (10, 50 μg/mL) for 16 h. Apoptotic and dead cells were identified by multiparameter flow-cytometry with annexin V–APC/propidium iodide (PI) staining.

### Target Next Generation Sequencing (NGS)

Genetic screening on the index patient was performed by Targeted NGS with custom target enrichment to identify variants in 314 PIDD-related genes. The assay was performed as previously described (17). The novel *FAS* heterozygous variant identified with this platform was confirmed by Sanger sequencing.

### Plasma Cytokine Levels and Luciferase Immunoprecipitation System (LIPS) for Anti-lymphocytes Antibodies

Biomarkers were measured by electrochemiluminescence with custom multiplex-kit (Meso Scale Discovery). sFAS plasma levels were measured by ELISA following the manufacturer's instructions (R&D Systems), Vitamin B12 levels were obtained by reviewing medical and laboratory records. Plasma samples were also screened for anti-CD4, anti-CTL4, anti-IL2RG, and anti-IL7R autoantibodies using a particle-based approach (LIPS) according to a previously described protocol (18).

### NK-Mediated ADCC Assay

NK cells were isolated from healthy control buffy coats using EasySep™ Human NK Cell Isolation Kit (Stemcell Technologies). The isolated NK-cells were incubated at 37°C overnight with 1,000 U/mL of IL-2 (Peprotech). From the same healthy control buffy coat, PBMCs were saved to be used as targets and incubated at 37°C overnight. The next day, the target cells were labeled with 0.6 μM of Carboxyfluorescein-succinimidyl-ester (CFSE). The cells were incubated at room temperature with 100 μl of PBS, anti-CD20, health control plasma, or patient plasma for 30 min, then washed. The targets cells were plated in a U-bottom 96-well plate with 10,000 cells/well. The NK-cells were plated with the described E:T ratios. The NK-cells and target cells were incubated for 4 h, then stained for CD3, CD4, CD8, and CD19 and analyzed by flow-cytometry. Each experimental condition had 2 replicates and cells were enumerated using counting-beads (ThermoFisher). Protein A/G affinity resin (ThermoFisher) was used for experimental condition in which depletion of total IgG was required. The % of killing was calculated as the difference in the absolute number of CFSE-labeled targets incubated with and without NK. Uptrending % of killing with increasing E:T ratio and reaching >20% at E:T ratio of 100:1 was considered consistent with specific NK-mediated ADCC.

### Statistical Analysis

Continuous variables were summarized as median (interquartile range) or average (standard error of the mean), whereas *n* (%) was used for categorical ones. Differences among groups were analyzed by Mann-Whitney test for unpaired analyses.

## Results

### Case Report

A 33-year-old female developed a disseminated vesicular rash on trunk and extremities with 40–50 non-synchronously evolving lesions, 2 weeks after receiving post-partum attenuated varicella vaccine. All lesions eventually crusted and resolved within 10 days from onset without any other clinical complications. Splenomegaly was noted on physical exam; it was previously documented in her physical exams since age 5 and was considered to be a sequela of infectious mononucleosis. With the exception of recurrent mild upper respiratory infections, she did not have any history of specific health complaints, hospitalizations, infectious complications, or lymphoadenopathy.

**Figure 1:**
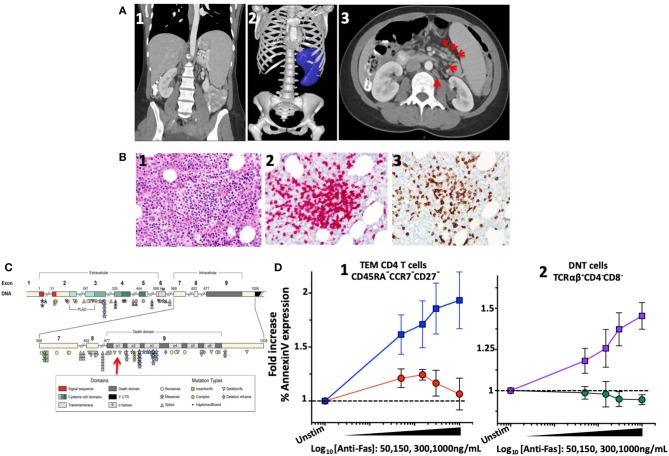
Imaging, bone marrow biopsy findings and characterization and location of the novel *FAS* heterozygous variant in a patient with CD4 lymphopenia and ALPS. **(A)** Computed tomography of abdomen and pelvis. (1) Coronal section: Splenomegaly. (2) Splenic volume estimation by 3D reconstruction: 1,034 milliliter (mL) (normal 200 ± 50 mL). (3) Axial section: Paraortic and mesenteric lymphadenopathy. **(B)** Bone marrow biopsy showing scattered lymphocytic aggregates with prevalence of CD8^+^ cells. (1) Lymphocytic aggregate in hematoxylin and eosin staining at 200x magnification. (2) CD3 Immunohistochemistry (IHC) staining. (3) CD8 IHC staining. **(C)** Diagram of the *FAS* gene identifying intron/exon structure and protein domains. Mutations are indicated at their approximate location and identified by symbols corresponding to mutation type. The new mutation identified in the index patient indicated by the red arrow. Figure adapted by Hsu et al. (3). **(D)** FAS-induced apoptosis was evaluated in terminal effector memory cells (TEM) and TCRαβ^+^ DN T cells of index patient and healthy subjects using an *ex vivo* flow cytometric assay based on FAS crosslinking. (1) Average fold increase ± standard error of mean (SEM) of Annexin-V expression in TEM (CD45RA^−^CCR7^−^CD27^−^) from 4 independent experiments with index patient (red) and 8 different healthy controls (blue) with different concentrations of anti-FAS crosslinking antibody. (2) Average fold increase ± SEM of Annexin-V expression in DNT cells (TCRαβ^+^CD4^−^CD8^−^) from 4 independent experiments with index patient (green) and 8 different healthy controls (purple) with different concentrations of anti-FAS crosslinking antibody.

The concern for varicella zoster virus (VZV) vaccine strain disseminated infection and the persistent leucopenia led to an initial work-up, which revealed selective IgA deficiency and CD4 lymphopenia (111–118 cells/μL). A presumed diagnosis of Idiopathic CD4 lymphopenia (ICL) was formulated, and she was referred to the NIH Clinical Center for further evaluation. Imaging confirmed splenomegaly (17 cm cranio-caudal length) and revealed modest mesenteric and retroperitoneal adenopathy (Figure 1A). Flow cytometric analysis of T-cell subpopulations confirmed CD4 lymphopenia (CD4 189 cells/μL), normal numbers of CD8 T cells (383 cells/μL), and an increased proportion and absolute number of CD3^+^TCRαβ^+^CD4^−^CD8^−^ double negative T cells (DNT) (4.3%, 33 cells/μL). In addition, high proportion of cycling of CD4 T-cells and DNT (Ki67 12 and 42%, respectively) as well as increased PD-1 expression in CD4 T-cells (42%) were found. The proportion and absolute number of total B cells were within normal range (CD19^+^: 16.4%, 75 cells/μL). Total IgG levels were moderately elevated (1,716 mg/dL), IgM were within normal range (54 mg/dL), while IgA undetectable. Bone marrow biopsy showed scattered lymphocytic aggregates with prevalence of CD8^+^ cells, consistent with previously reported findings in bone marrow biopsy of patients with ALPS (19) (Figure 1B). Targeted NGS with custom target enrichment revealed a novel heterozygous missense variant in the death domain (NM_000043 c.719T>A p.M240K) of the *FAS* gene and was confirmed by direct Sanger sequencing (Figure 1D).

FAS-induced apoptosis was evaluated in a crosslinking assay to determine the functional effect of such novel genetic variant (13). Apoptosis was expressed as normalized fold-change of Annexin-V expression at different concentrations of crosslinking FAS antibody. In effector memory CD4 T-cells (CD3^+^CD4^+^CD45RA^−^CCR7^−^CD27^−^) from healthy individuals (*n* = 8), an average increase of 1.93 ± 0.2-fold increase in Annexin-V expression was found with 1,000 ng/mL of crosslinking FAS antibody. At the same concentration of crosslinking FAS antibody (1,000 ng/mL), the Annexin-V expression remained unchanged in the index patient (1.06 ± 0.1-fold, *n* = 4, *p* = 0.01) (Figure 1C). Similarly, while Annexin-V expression increased significantly in DNT TCRαβ^+^ T-lymphocytes of healthy individuals (1.57 ± 0.07 with 1,000 ng/mL, *n* = 8), it remained unchanged in the index patient (0.94 ± 0.03-fold, *n* = 4, *p* = 0.0002).

**Figure 2:**
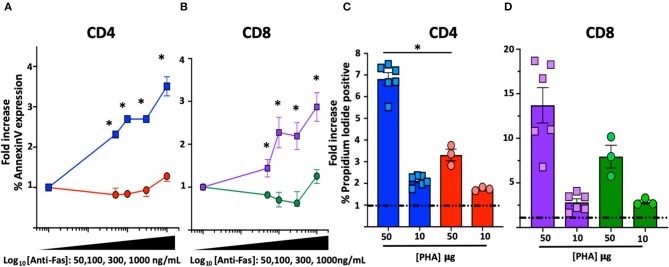
FAS-induced apoptosis and activation-induced-cell death (AICD) in T cell blasts from the index patient and healthy subjects. *Comparisons with statistically significant differences. **(A)** Average fold increase ± SEM of Annexin-V expression in CD4 T cell blasts from index patient (red) and 2 different healthy controls (blue) with different concentrations of anti-FAS crosslinking antibody. **(B)** Average fold increase ± SEM of Annexin-V expression in CD8 T cell blasts from index patient (green) and 2 different healthy controls (purple) with different concentrations of anti-FAS crosslinking antibody. **(C)** Average fold increase ± SEM in Propidium Iodide (PI) positive CD4 T cell blasts from index patient (red) and 2 different healthy controls (blue) with 2 different concentrations of PHA. **(D)** Average fold increase ± SEM in PI positive CD8 T cells blasts from index patient (green) and 2 different healthy controls (purple) with 2 different concentrations of PHA.

Functional characterization of the novel FAS genetic variant also included FAS-induced apoptosis and sensitivity to activation-induced-cell-death (AICD) assays on T cell blasts (14, 15). A significant reduction of FAS-induced apoptosis was found in T cell blasts from the index patient at all concentration of crosslinking FAS antibody: at the highest concentration (1,000 mg/mL), Annexin-V expression increased in CD4 (3.06 ± 0.2-fold, *n* = 6) and CD8 (2.8 ± 0.3-fold, *n* = 6) blasts from healthy individuals but remained substantially unchanged in CD4 (1.2 ± 0.05-fold, *n* = 3, *p* = 0.02) and CD8 (1.2 ± 0.1-fold, *n* = 3, *p* = 0.02) blasts from the index patient (Figures 2A,B). Similarly, a significant reduction in AICD was found in T cell blasts of the index patient: the proportion of dead cells after stimulation with the highest concentration of PHA (50 μg/mL) increased in CD4 (6.8 ± 0.3-fold, *n* = 6) and CD8 (13.7 ± 2-fold, *n* = 6) blasts from healthy individuals but to a lower extent in CD4 (3.3 ± 0.25-fold, *n* = 3, *p* = 0.02) and CD8 (8 ± 1.2-fold, *n* = 3, *p* = 0.09) blasts from the index patient (Figures 2C,D).

Further immunological evaluation of the patient included measurement of plasma cytokines and showed an increased level of soluble FAS ligand (801 pg/mL, normal level < 200 pg/mL) and increased levels of Interleukin-18 (7,248 pg/mL). A definitive diagnosis of ALPS-FAS was formulated based on the 2010 revised diagnostic criteria as both required criteria were satisfied (chronic lymphadenopathy, splenomegaly, and increased TCRαβ+ DNT cells), in addition to one primary criterion (pathogenic germline mutation in *FAS* death domain).

### CD4 Lymphopenia in a Cohort of Patients With ALPS-FAS

ALPS-FAS has not been previously associated with CD4 lymphopenia and such finding in our index patient was unexpected and counterintuitive in a disorder of FAS-mediated apoptosis, which is usually associated with lymphocytosis and lymphoproliferation. For this reason, we conducted a retrospective analysis in 169 patients enrolled in the NIH ALPS-FAS cohort and documented CD4 lymphopenia (< 300 cells/μL in absence of any steroid or immunomodulant treatment) in 8 patients (4.7%, Table 1) including the above described index patient. These cases were matched 2:1 by age (adult or pediatric), site, and type of genetic variant (death domain, extracellular or transmembrane domain; germline or somatic) with patients with CD4> 300 T-cells/μL and confirmed diagnosis of ALPS-FAS. The laboratory records median CD4 cell counts in cases was 253 cells/μL (25%) and 774 (39%) in controls. No significant differences were found between cases and controls in the prevalence of autoimmune cytopenias (immune thrombocytopenic purpura, autoimmune hemolytic anemia, autoimmune neutropenia) or a panel of clinical autoantibodies including anti-thyroglobulin, anti-thyroperoxidase, extractable nuclear antigen (ENA), antinuclear antibodies (ANA), rheumatoid factor (RF), anticardiolipin (ACA), anti-neutrophil cytoplasmic (ANCA), or lupus anticoagulant (*p* > 0.1). Splenomegaly was noted in 75% of cases (6 out of 8) and 69 % of controls (11 out of 16). Five cases (62.5%) and their matched 10 controls had a germline pathogenic mutation in the *FAS* death domain, 1 case and its 2 matched controls had a somatic mutation in the *FAS* death domain in TCRαβ^+^ DNT, while the remaining 2 cases and matched controls had a germline mutation in the extracellular domain and in the transmembrane domain, respectively.

### Immunophenotyping in Patient With ALPS-FAS With or Without CD4 Lymphopenia

**Figure 3:**
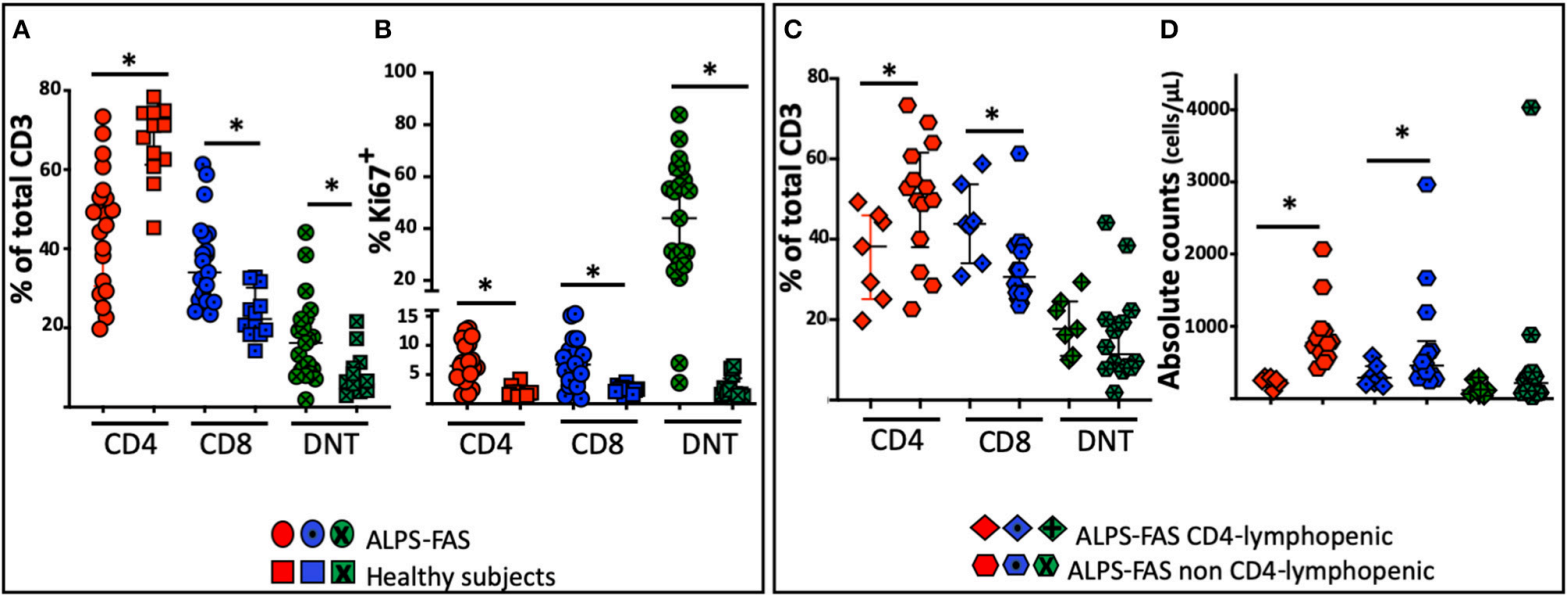
Distribution of the proportion of CD4, CD8, and DNT-cells in patient with ALPS-FAS and CD4 lymphopenia, matched controls subjects with ALPS-FAS and healthy subjects. ^*^Comparisons with statistically significant differences. **(A)** Proportion of CD4, CD8, and DNT cells in patients with ALPS-FAS (circles) and healthy subjects (squares). **(B)** Proportion of Ki67 expressing CD4, CD8, and DNT cells in patients with ALPS-FAS (circles) and healthy subjects (squares). **(C)** Proportion of CD4, CD8, and DNT-cells in ALPS-FAS patients with (diamonds) or without (hexagons) CD4 lymphopenia. **(D)** Absolute counts of CD4, CD8, and DNT-cells in ALPS-FAS patients with (diamonds) or without (hexagons) CD4 lymphopenia.

As expected, differences in the distribution of T cell subpopulation were noted between patients with ALPS-FAS irrespectively of absolute CD4 T-cells counts and healthy individuals (HC): we found a reduced proportion of CD4 T-cells [median 70% (IQR: 61–74), *n* = 12 in HC vs. 49% (IQR: 62–75), *n* = 21 in patient with ALPS, *p* < 0.0001] associated with a relative increased proportion of DN T-cells [median 6.5% (IQR: 4.5–11) *n* = 12 in HC vs. 16.2% (IQR: 9–22), *n* = 21 in patients with ALPS-FAS, *p* = 0.006] and a reduction of CD8-T cells [median 22% (IQR: 18–31) *n* = 12 in HC vs. 34% (IQR: 37–43), *n* = 21 in patients with ALPS-FAS, *p* < 0.0003] (Figure 3A).

As previously described, a large proportion of the expanded population of TCRαβ^+^ DNT-cells present in ALPS-FAS patients also expressed the B-cell-specific CD45R isoform B220 [median 10% (IQR: 2–19) *n* = 3 in HC vs. 57% (IQR: 43–72), *n* = 21 in patient with ALPS-FAS, *p* < 0.0001].

No significant changes were observed in the proportion of naïve and memory CD4 or CD8 subpopulations or in the proportion of regulatory T cells or CD95 expressing CD4, CD8 or DNT-cells (*p* > 0.05). Patients with ALPS-FAS, however, were found to have a slightly lower level of expression of CD127 on CD4 T-cells [median 90.7% (IQR: 90.5–92), *n* = 3 in HC vs. 86.4% (IQR: 78–88), *n* = 21 in patients with ALPS-FAS, *p* < 0.003] but not on CD8 T-cells [72% (IQR: 70.5−85, *n* = 3) in HC vs. 61 (IQR: 54–75.5%, *n* = 21) in patient with ALPS-FAS, *p* > 0.1)]. Moreover, in patients with ALPS-FAS, we found a higher level of expression of Ki67 in CD4, CD8, and DNT-cells compared to healthy individuals (median 6.5, 6, 44%, in CD4, CD8, and DNT of ALPS-FAS patients vs. 1.8, 2.4, 1.7% in CD4, CD8, and DNT of HC, *p* < 0.0001, *p* = 0.001, *p* < 0.0001, respectively) (Figure 3B).

In an attempt to identify immunological correlates and/or underlying mechanisms for CD4 lymphopenia in ALPS-FAS, we compared the immunophenotypic characteristics and plasma cytokine levels of patients with ALPS-FAS with or without CD4 T-cells lymphopenia. The immunophenotypic analysis confirmed a lower percentage of CD4 T-cells [median 38% (IQR: 25–45) vs. 51% (IQR: 34–53), *n* = 7, and *n* = 14, respectively, *p* = 0.02] associated with a relative increase in the proportion of CD8 [median 44% (IQR: 34–54) vs. 30% (IQR: 26–38), *n* = 7, and *n* = 14, respectively, *p* = 0.01] and no differences in the proportion of DNT in CD4 lymphopenic ALPS-FAS patients compared to matched control ALPS-FAS patients without CD4 T-cells lymphopenia (Figure 3C). The absolute counts reflected the changes observed in the proportion of the T cell subsets of ALPS-FAS patients with or without CD4 T-cells lymphopenia: CD4 [median 257 (IQR: 216–294) vs. 774 (IQR: 624–946) cells/μL, *p* < 0.0001], CD8 [median 290 (IQR: 203–450) vs. 459 (IQR: 301–796) cells/μL, *p* = 0.046], DNT [median 119 (IQR: 67–268) vs. 216 (IQR: 83–358) cells/μL, *p* = 0.25] (Figure 3D). The proportion and the absolute counts of CD4 and DNT were correlated in patients with ALPS, negatively for the proportion [Pearson *r*: −0.67, *p* = 0008] and positively for the absolute counts [Pearson *r*: 0.71, *p* = 0003]. Such correlations between CD4 and DNT proportion and absolute counts were maintained in patients with ALPS without CD4 lymphopenia (Pearson *r*: −0.74, *p* = 0.002, and 0.76, *p* = 0.0008, respectively) but were absent in patients with ALPS with CD4 lymphopenia (Supplemental Table 1).

No significant differences in the proportion of naïve, central memory, effector memory, and terminal effector memory CD4 and CD8 T-cells was noted among patients with ALPS-FAS with or without CD4 lymphopenia (*p* > 0.05). The proportion of recent thymic emigrant CD4 T cells (CD45RA^+^CD27^+^CD31^+^) did not differ in the 2 groups [median 18% (IQR: 10–24) vs. 18% (IQR: 14–25), *n* = 7, and *n* = 14, respectively, *p* < 0.1].

**Figure 4:**
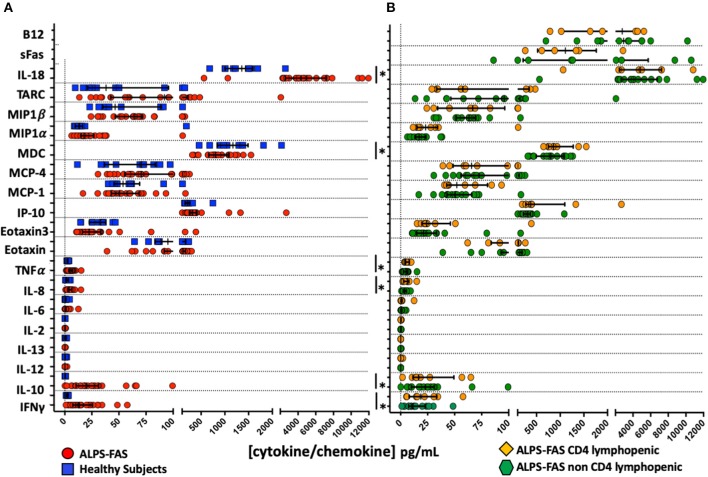
Plasma concentration of cytokines and chemokines involved in inflammatory responses and regulation of T cell function. ^*^Comparisons with statistically significant differences. **(A)** Plasma concentration of cytokines and chemokines in patients with ALPS-FAS (red circles) and healthy subjects (blue squares). **(B)** Plasma concentration of cytokines and chemokines in patients with ALPS-FAS with (orange diamonds) or without (green exagons) CD4 lymphopenia.

Several significant changes were noted in plasma cytokines levels of patient with ALPS-FAS compared to HC: increased concentrations of IFNγ (median 13.2 vs. 2.5 pg/mL, *p* = 0.002), IL-10 (19.9 vs. 0.336 pg/mL, *p* = 0.002), IL-8 (4 vs. 1.6 pg/mL, *p* = 0.01), TNFα (4.8 vs. 2.5 pg/mL, *p* = 0.007), IL-18 (4,348 vs. 1,357 pg/mL, *p* = 0.001) and decreased levels of MDC (804 vs. 1,170 pg/mL) (Figure 4A). In contrast, no significant differences were found in the level of 22 cytokines involved in inflammatory responses, T-cell activation and proliferation between patients with ALPS-FAS with or without decreased number of CD4 T-cells (Figure 4B).

### Anti-lymphocyte Autoantibodies in ALPS-FAS

Since autoimmune cytopenias are among the most typical clinical features of ALPS-FAS, we hypothesized that the reduced number of CD4 cell counts could be secondary to autoimmune destruction of CD4 T-cells, impaired homeostatic expansion or altered trafficking due to autoantibodies.

To further elucidate anti-lymphocyte auto-antibody production, we preformed LIPS to detect autoantibodies against CD4, CTLA4, IL-7, receptor or IL-2γ-chain. LIPS is based on the fusion of protein antigen to a light-emitting enzyme reporter and represents a powerful and sensitive method to identify autoantibodies (18). No evidence of autoantibodies where found in any of the patient with ALPS-FAS, but the absence of autoantibodies against these 4 specific targets by LIPS, would not completely eliminate the possibility of autoantibodies against other epitopes expressed on the surface of CD4 T-cells.

**Figure 5:**
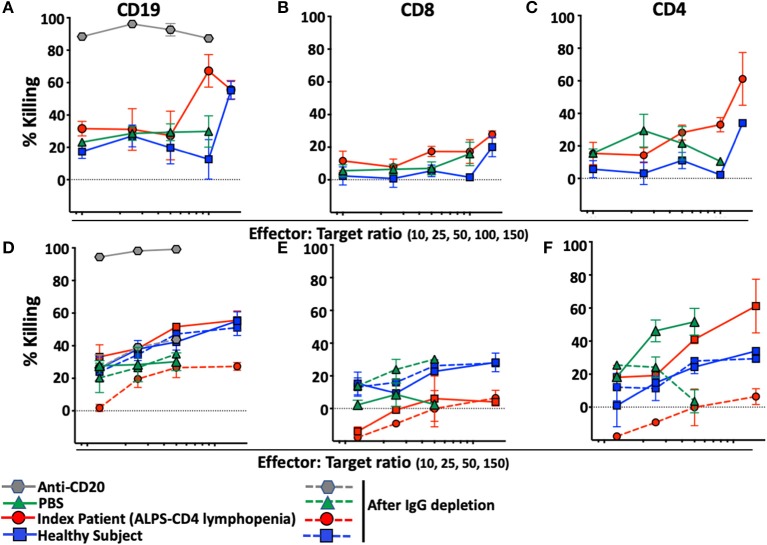
NK-mediated antibody dependent direct cytotoxicity of CD19, CD4, or CD8 T-cells in the index patient with ALPS-FAS and CD4 lymphopenia. Killing is expressed as percentage of untreated control. CSFE-stained healthy donor cells after incubation at different E:T ratio with autologous NK cells in presence of monoclonal antibody Anti-CD20 (positive control for NK mediated ADCC, gray hexagon), PBS (green triangle), and plasma from healthy control (blue square) or index patient (red circle) with ALPS and CD4 lymphopenia. **(A–C)** NK-mediated killing of CD19 B-cells, CD8 T-cells, CD4 T-cells, respectively. Presented is average ± standard error of the mean (SEM) of 3 independent experiments each with 2 replicates per experimental condition. **(D–F)** NK-mediated killing of CD19 B-cells, CD8 T-cells, CD4 T-cells before and after incubation with Protein A/G agarose affinity resin for depletion of IgG in each experimental condition. Presented is average ± SEM of 2 replicates per experimental condition.

To determine whether the presence of anti-lymphocyte autoantibodies caused depletion of lymphocytes, we developed an assay designed to reveal NK-cell-mediated antibody-dependent-cellular-cytotoxicity (ADCC) against B or T-lymphocytes. The assay is based on the use of target cells (NK-depleted PBMC including CD19, CD8, or CD4) stained with Carboxyfluorescein-succinimidyl-ester (CFSE), incubated with autologous NK cells purified from the same healthy donor in the presence of plasma of interest. As expected, rituximab, a monoclonal anti-CD20 antibody, causes massive depletion of B-cells in this assay (Figure 5A) and is used as positive control for NK-mediated ADCC. No CD8 T-cell depletion was observed with PBS, plasma from a healthy control donor or plasma from our index patient with ALPS-FAS and CD4 lymphopenia (Figure 5B). In contrast, CD4 T-cells were specifically depleted when incubated with plasma from our index patient but not with PBS or plasma from a healthy donor (Figure 5C). Increasing Effector:Target (E:T) ratio resulted in more prominent NK-mediated killing of CD4 T-cells up to an average level of 60% depletion of target CD4 T-cells at an E:T ratio of 150:1. Moreover, NK-mediated killing of CD4 T-cells was abrogated by the depletion of plasma IgG (Figures 5D–F).

**Figure 6:**
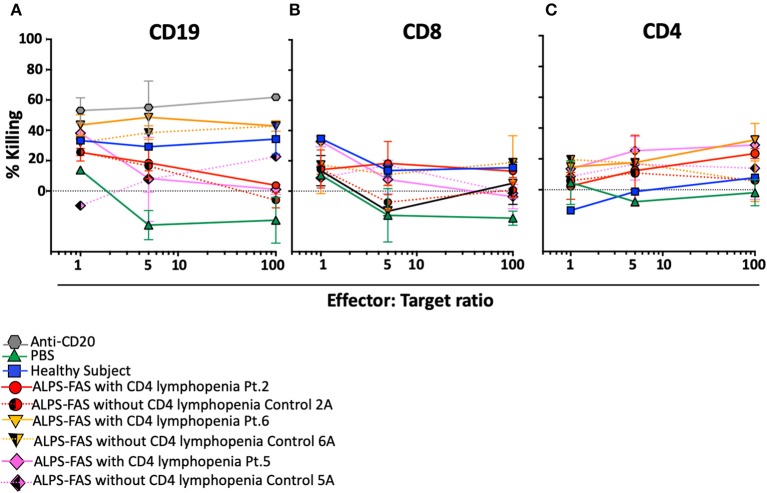
NK-mediated antibody dependent direct cytotoxicity of CD19, CD4, or CD8 T cells in the patients with ALPS with or without CD4 lymphopenia. Killing is expressed as percentage of untreated control. CSFE stained after incubation at 4 different E:T ratio (1, 5, 50, 100) with autologous NK cells in presence of monoclonal antibody Anti-CD20 (positive control for NK mediated ADCC, gray hexagon), PBS (green triangle), and plasma from healthy control (blue square), ALPS-FAS patients with CD4 lymphopenia (red circle, orange triangle, pink diamond, continuous lines) and relative matched controls with ALPS-FAS and no evidence of CD4 lymphopenia (hatched red circle, hatched orange triangle, hatched pink diamond, dotted lines). **(A)** NK-mediated ADCC killing of CD19 B cells. **(B)** NK-mediated ADCC killing of CD8 T cells. **(C)** NK-mediated ADCC killing of CD4 T-cells.

Plasma of the 4 additional patients with ALPS-FAS and CD4 lymphopenia was tested in the NK mediated ADCC assay: in 3 of the 7 subjects we documented uptrending specific killing of CD4 T-cells at increasing E:T ratio reaching >20% at 100:1 E:T ratio (Patient 2: average 23 ± 2%; Patient 6: 32 ± 7%; Patient 5: 29 ± 0.3%), while matched control plasma from all ALPS-FAS patients without CD4 lymphopenia (Control 2: average 6 ± 1%; Control 6: 5 ± 9%; Patient 5: 13 ± 14%, *p* = 0.01), and plasma from healthy subjects (average 7 ± 1%) did not significantly affect CD4 T-cells number and did not have evidence of uptrending specific killing activity with increasing E:T ratio (Figures 6A–C). No killing of CD8 T-cells was observed.

**Figure 7:**
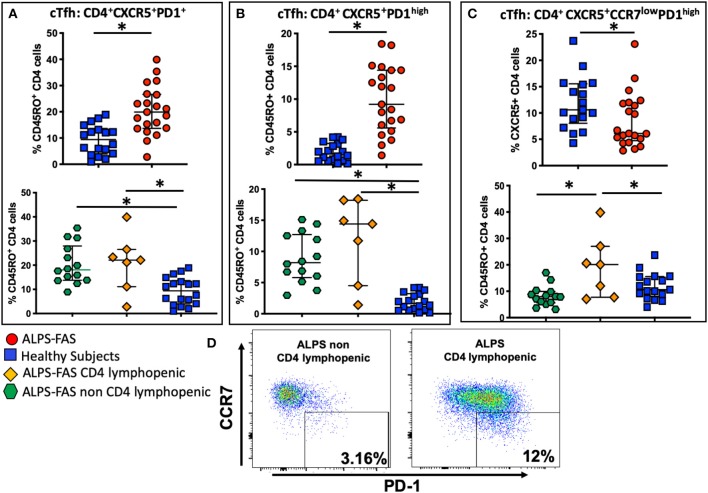
Circulating T follicular helper cells (cTfh) and theirs subpopulation in health subjects (blue square), ALPS-FAS subjects irrespective of CD4 cell counts (red circles), ALPS-FAS with CD4 lymphopenia (orange diamond), and ALPS-FAS without CD4 lymphopenia (green hexagon). ^*^Comparisons with statistically significant differences. **(A)** Proportion of total cTfh cells (CD4^+^CD45RO^+^CXCR5^+^). **(B)** Proportion of activated Tfh (CD4^+^CD45RO^+^CXCR5^+^PD1^high^). **(C)** Proportion of a CCR7^low^ expressing cTfh (CD4^+^CD45RO^+^CXCR5^+^CCR7^low^PD1^high^). **(D)** Representative scatter dot plot flow-cytometry panel highlighting the expansion of the CCR7^low^PD1^high^ Tfh subset in PBMC from patient with ALPS-FAS with or without CD4 lymphopenia.

Recently, it has been documented that ALPS-FAS is also characterized by an enriched population of unique germinal center (GC) B cells that drive the production of autoantibodies (20), however, the cells that regulate GC B-cells dynamics, T-follicular-helper-cells (Tfh), have not yet been implicated in the pathogenesis in autoimmune phenomena in ALPS-FAS. We evaluated the proportion and phenotype of circulating Tfh (cTfh) as identified by the expression of CXCR5, the chemokine receptor that specifically determines trafficking of lymphocytes to the GC. Within the CD45RO^+^CD4 T-cells compartment, ALPS-FAS patients had a higher proportion of cTfh (CXCR5^+^PD-1^+^) when compared to HC [median 19.9% (IQR: 13.7–26.65) vs. 9.4% (IQR: 4–13.6), *p* < 0.0001, respectively] (Figure 7A) and also an elevated proportion of highly activated CXCR5^+^PD-1^high^ cTfh when compared to HC [median 9.2% (IQR: 5.59 − 14.4) vs. 1.6% (IQR: 0.6–3.2), *p* ≤ 0.0001)] (Figure 7B). No significant differences were identified in the proportion of cTfh or highly activated cTfh in ALPS-FAS patient with or without CD4 lymphopenia (Figures 7A,B); however, a particular subpopulation of cTfh, CXCR5^+^CD4^+^CCR7^low^PD-1^high^, which has been highly correlated with autoantibody production in rheumatoid arthritis and SLE (11), was largely expanded in ALPS-FAS CD4 lymphopenic patients compared to ALPS-FAS patients without CD4 lymphopenia [median 20.1% (IQR: 7.73–27) vs. 7.94% (IQR: 5.61–9.93), *p* = 0.025)] (Figures 7C,D). The absolute number of cTFH and all their subsets reflected the absolute number of CD4 T cells and was significantly lower in the ALPS-FAS patients with CD4 lymphopenia compared to ALPS-FAS patients without CD4 lymphopenia [cTFH (CXCR5^+^PD-1^+^: median 18.2 cells/μL (IQR: 12.8–35) vs. 57 cells/μL (IQR: 43.6–83.5), *p* = 0.003)], highly activated [TFH (CXCR5^+^PD-1^high^: median 10.7 cells/μL (IQR: 5.2–18.1) vs. 28.4 cells/μL (IQR: 18.1–40.4), *p* = 0.007)]), and [CD4^+^CXCR5^+^CCR7^low^PD-1^high^: median 1.8 cells/μL (IQR: 1.5–2.5) vs. 3.7 cells/μL (IQR: 2–7), *p* = 0.03), respectively].

## Discussion

Herein, we report for the first time on the paradoxical occurrence of CD4 lymphopenia in the context of ALPS-FAS, a heterogeneous genetic disorder of lymphocyte homeostasis dominated by benign lymphoproliferation and autoimmune phenomena (6). We found that 4.7% (8 out of 170) of patients with ALPS-FAS in the NIH cohort had CD4 T-cell counts below 300 cells/μL regardless of age and exact location of underlying FAS genetic defect. It is unlikely that sequestration in an enlarged spleen and/or in other lymphoid tissue in the reticuloendothelial system may entirely explain the development of CD4 lymphopenia in ALPS-FAS as hypersplenism and lymphadenopathy are present in the majority of patients, while CD4 lymphopenia is observed only in a small subset. Therefore, we speculated that CD4 lymphopenia in ALPS-FAS could be caused by (a) decreased thymic output, (b) reduced homeostatic proliferation in paracortical T cell areas engulfed with DNT-cells, or (c) autoimmune peripheral destruction. We did not find any difference in the proportion of recent thymic emigrants as measured by expression of CD31 on naïve CD4 T-cells nor any significant change in the proportions of naïve or memory CD4 T cells, or in the proportion of cycling CD4 T cells in the CD4 lymphopenic patients with ALPS-FAS compared to matched controls with ALPS-FAS and normal CD4 cell counts. We could document several immunological features specifically associated with ALPS and the profound dysregulation secondary to impaired apoptosis, including the accumulation of cycling DNT-cells, ALPS-FAS-specific phenotypic features (CD45R B220 expression on DNT-cells) as well as the changes in cytokines involved in the regulation of T-cells function and inflammation (IL-10, IL-8, TNFα, IL-18, and decreased MDC). Although we identified some novel changes in biomarkers not previously associated with ALPS (increased IFNγ, decreased MDC) (21), we did not initially identify any specific change in biomarkers or intrinsic immunophenotypic features of CD4 T-cells that was associated with the reduced number of CD4 T-cells in ALPS-FAS (4, 21, 22).

The lack of any evidence supporting reduced thymic output or altered T-cells peripheral homeostasis along with a homogenous loss of CD4 T-cells of either naïve or memory phenotype in a disease typically associated with accumulation of autoreactive T cells and autoimmune phenomena, led us to consider an autoimmune pathogenesis for the reduced CD4 T-cells observed in some patients with ALPS-FAS. Although we did not identify autoantibodies specifically targeting the CD4 molecule, we used an NK-mediated-ADCC assay to reveal the presence of autoantibodies on the surface of CD4 T-cells. We found a remarkable correspondence between the *in vitro* ADCC mediated lysis of CD4 T-cells by plasma of some patients with ALPS-FAS and *in vivo* CD4 lymphopenia. In fact, plasma of 4 of the 8 subjects, including our index patient, was effective in mediating ADCC of CD4 T-cells and not of CD8. The evidence of specific IgG-mediated ADCC of CD4 T-cells from different healthy donors only in CD4 lymphopenic patients with ALPS-FAS argues in favor of the presence of anti-lymphocyte antibodies targeting epitopes expressed on the surface of CD4 T-cells. Moreover, it is plausible that, such anti-lymphocytes autoantibodies, can also mediate the depletion of CD4-T-cells through other mechanisms of antibody-mediated killing [i.e., complement mediated cytotoxicity (CDC) or antibody-mediated phagocytosis (ADph)] (23). Therefore, considering that the accumulation of autoreactive lymphocytes and the consequent loss of peripheral tolerance observed in ALPS-FAS is often associated with autoimmune hemolytic anemia, thrombocytopenia and neutropenia, it is not entirely surprising that autoimmune CD4 lymphopenia may occur in such immunological context.

The evidence of autoantibodies against CD4-T-cells in ALPS-FAS led us to further characterize the phenotypic and functional features of Tfh in such immunological context, as this T-cell subsets is a key regulator of the B-cell function (24) that has never been previously investigated in patients with ALPS-FAS. An animal model showed that C57BL/6 *FAS* deficient mice have increased frequencies of Tfh and Tfh-associated effector cytokines compared to their C57BL/6 wildtype counterpart and develop higher serum autoantibodies and enhanced splenic B-cell activation (25), possibly suggesting a role for Tfh in the production of autoantibodies. Here, in the first evaluation of Tfh in human subjects with ALPS, we found that ALPS-FAS is associated with an expansion of the proportion of total cTfh and activated cTfh compared to healthy individuals. Strikingly, in addition to this novel general immunological feature of ALPS-FAS, we found that only ALPS-FAS CD4 lymphopenic subjects also had an expansion of the proportion of specific subset of cTfh (CCR7^low^PD-1^high^). Such subset has been already associated with autoantibody production in other models of autoimmune diseases (11) and our observation suggest that perhaps, in ALPS-FAS the dysregulated differentiation of this specific subset of cTfh resulted in selection of autoreactive B cells and consequent autoantibody production as suggested in rheumatoid arthritis and SLE (11). The increase in the proportion of CD4^+^CXCR5^+^CCR7^low^PD-1^high^ cTFH in CD4-lymphopenic ALPS patients was associated with a reduced absolute number of these cells compared to non-CD4-lymphopenic ALPS patients, reflecting the differences in the total CD4 T cells between these 2 groups. However, irrespective of the absolute counts of CD4^+^CXCR5^+^CCR7^low^PD-1^high^ cTFH, the increase in their proportion compared to other cTFH subsets reflects an aberrant distribution of these subsets consistent with the functional pattern of an active Tfh differentiation in lymphoid organs that correlates with clinical indices in autoimmune diseases. Furthermore, autoimmune cytopenias (i.e., neutropenia, anemia, or thrombocytopenia) often cause morbidity not only in ALPS-FAS and other autoimmune diseases but also in other PIDDs [i.e., CTLA4-deficiency, Lipopolysaccharide-Responsive vesicle trafficking Beige-like and Anchor protein (LRBA) defects, STAT-3 gain of function, CVID, activated-PI3Kδ syndrome (APDS)] (26–29) and it is plausible that altered number and or function of cTfh is associated with production of auto-antibodies in other diseases characterized by disturbed T-cell and B-cell interaction and regulation as recently shown in APDS (30) and LRBA deficiency (31).

Irrespectively of the mechanisms underlying CD4 lymphopenia, we suggest that the presence of CD4 lymphopenia in the context of ALPS-FAS may have clinical implications for its diagnosis, natural history and treatment. As seen in our index patient, a clinical presentation with an atypical course of viral infection and CD4 lymphopenia in presence of splenomegaly should prompt a genetic work-up aimed to rule out atypical presentation of primary immune deficiencies in general and ALPS in particular, before a diagnosis of ICL is considered. It is unclear whether low CD4 cell counts in the context of ALPS-FAS may increase the risk for opportunistic infection but specific clinical monitoring or targeted prophylactic intervention or immunization strategies may be considered in these patients, especially when immunomodulant intervention for treatment of ALPS-FAS is being adopted. Despite the limitation of the small number of patients, the retrospective nature of our work and the lack of histological evaluations of lymphoid tissue, we identified a subgroup of patient with ALPS-FAS with unexpected CD4 lymphopenia caused by anti-lymphocyte autoantibodies mediating ADCC of CD4 T-cells and associated with the expansion of a specific subset of cTfh (CXCR5^+^CCR7^low^PD-1^high^) already linked to autoantibody production in other clinical contexts. This novel mechanism of autoimmune lymphopenia leading to CD4 T-cell depletion may have implication for the clinical management of patients with ALPS-FAS as well as other PIDDs and autoimmune diseases.

## Ethics Statement

All patients provided written informed consent prior to study entry under NIH Clinical Center institutional review board-approved protocols (https://clinicaltrials.gov/ct2/show/NCT00001350; https://clinicaltrials.gov/ct2/show/NCT00867269).

## Author Contributions

AL, IS, and VR designed research and wrote the paper. AL, C-SW, PY, JN, ER, HM, MS, BL, JS, SR, CV, AR, IM, AP-D, and PB performed research and analyzed data. AL, SP, MA, MM, DP, VR, and IS provided clinical care to enrolled patients.

### Conflict of Interest Statement

AR is employed by the biomedical company Leidos Biomedical Research. The remaining authors declare that the research was conducted in the absence of any commercial or financial relationships that could be construed as a potential conflict of interest.

## References

[1] Medical Advisory Committee of the Immune Deficiency Foundation Shearer WT Fleisher TA Buckley RH Ballas Z Ballow M. Recommendations for live viral and bacterial vaccines in immunodeficient patients and their close contacts. J Allergy Clin Immunol. (2014) 133:961–6. 10.1016/j.jaci.2013.11.043 24582311 PMC4009347

[2] Fisher GH Rosenberg FJ Straus SE Dale JK Middleton LA Lin AY. Dominant interfering Fas gene mutations impair apoptosis in a human autoimmune lymphoproliferative syndrome. Cell. (1995) 81:935–46. 10.1016/0092-8674(95)90013-6 7540117

[3] Hsu AP Dowdell KC Davis J Niemela JE Anderson SM Shaw PA. Autoimmune lymphoproliferative syndrome due to FAS mutations outside the signal-transducing death domain: molecular mechanisms and clinical penetrance. Genet Med. (2012) 14:81–9. 10.1038/gim.0b013e3182310b7d 22237435

[4] Bleesing JJ Brown MR Straus SE Dale JK Siegel RM Johnson M. Immunophenotypic profiles in families with autoimmune lymphoproliferative syndrome. Blood. (2001) 98:2466–73. 10.1182/blood.V98.8.2466 11588044

[5] Rao VK Oliveira JB. How I treat autoimmune lymphoproliferative syndrome. Blood. (2011) 118:5741–51. 10.1182/blood-2011-07-325217 21885601 PMC3228494

[6] Price S Shaw PA Seitz A Joshi G Davis J Niemela JE. Natural history of autoimmune lymphoproliferative syndrome associated with FAS gene mutations. Blood. (2014) 123:1989–99. 10.1182/blood-2013-10-535393 24398331 PMC3968385

[7] Bousfiha A Jeddane L Picard C Ailal F Bobby Gaspar H Al-Herz W. The 2017 IUIS phenotypic classification for primary immunodeficiencies. J Clin Immunol. (2018) 38:129–43. 10.1007/s10875-017-0465-8 29226301 PMC5742599

[8] Bonilla FA Khan DA Ballas ZK Chinen J Frank MM Hsu JT. Practice parameter for the diagnosis and management of primary immunodeficiency. J Allergy Clin Immunol. (2015) 136:1186–205.e1–78. 10.1016/j.jaci.2015.04.049 26371839

[9] Picard C Bobby Gaspar H Al-Herz W Bousfiha A Casanova JL Chatila T. International union of immunological societies: 2017 primary immunodeficiency diseases committee report on inborn errors of immunity. J Clin Immunol. (2018) 38:96–128. 10.1007/s10875-017-0464-9 29226302 PMC5742601

[10] Zonios DI Falloon J Bennett JE Shaw PA Chaitt D Baseler MW. Idiopathic CD4+ lymphocytopenia: natural history and prognostic factors. Blood. (2008) 112:287–94. 10.1182/blood-2007-12-127878 18456875 PMC2442741

[11] He J Tsai LM Leong YA Hu X Ma CS Chevalier N. Circulating precursor CCR7(lo)PD-1(hi) CXCR5(+) CD4(+) T cells indicate Tfh cell activity and promote antibody responses upon antigen reexposure. Immunity. (2013) 39:770–81. 10.1016/j.immuni.2013.09.007 24138884

[12] Schulze-Koops H. Lymphopenia and autoimmune diseases. Arthritis Res Ther. (2004) 6:178–80. 10.1186/ar1208 15225363 PMC464928

[13] Lo B Ramaswamy M Davis J Price S Rao VK Siegel RM. A rapid *ex vivo* clinical diagnostic assay for fas receptor-induced T lymphocyte apoptosis. J Clin Immunol. (2013) 33:479–88. 10.1007/s10875-012-9811-z 23054345 PMC3567298

[14] Del-Rey M Ruiz-Contreras J Bosque A Calleja S Gomez-Rial J Roldan E. A homozygous Fas ligand gene mutation in a patient causes a new type of autoimmune lymphoproliferative syndrome. Blood. (2006) 108:1306–12. 10.1182/blood-2006-04-015776 16627752

[15] Del-Rey MJ Manzanares J Bosque A Aguiló JI Gómez-Rial J Roldan E. Autoimmune lymphoproliferative syndrome (ALPS) in a patient with a new germline Fas gene mutation. Immunobiology. (2007) 212:73–83. 10.1016/j.imbio.2006.12.003 17336828

[16] Martínez-Lorenzo MJ Alava MA Gamen S Kim KJ Chuntharapai A Piñeiro A. Involvement of APO2 ligand/TRAIL in activation-induced death of Jurkat and human peripheral blood T cells. Eur J Immunol. (1998) 28:2714– 10.1002/(SICI)1521-4141(199809)28:09&lt;2714::AID-IMMU2714&gt;3.0.CO;2-9 9754559

[17] Stoddard JL Niemela JE Fleisher TA Rosenzweig SD. Targeted NGS: a cost-effective approach to molecular diagnosis of PIDs. Front Immunol. (2014) 5:531. 10.3389/fimmu.2014.00531 25404929 PMC4217515

[18] Burbelo PD Lebovitz EE Notkins AL. Luciferase immunoprecipitation systems for measuring antibodies in autoimmune and infectious diseases. Transl Res. (2015) 165:325–35. 10.1016/j.trsl.2014.08.006 25241936 PMC4306608

[19] Xie Y Pittaluga S Price S Raffeld M Hahn J Jaffe ES. Bone marrow findings in autoimmune lymphoproliferative syndrome with germline FAS mutation. Haematologica. (2017) 102:364–72. 10.3324/haematol.2015.138081 27846610 PMC5286944

[20] Butt D Chan TD Bourne K Hermes JR Nguyen A Statham A. FAS Inactivation releases unconventional germinal center b cells that escape antigen control and drive IgE and autoantibody production. Immunity. (2015) 42:890–902. 10.1016/j.immuni.2015.04.010 25979420

[21] Caminha I Fleisher TA Hornung RL Dale JK Niemela JE Price S. Using biomarkers to predict the presence of FAS mutations in patients with features of the autoimmune lymphoproliferative syndrome. J Allergy Clin Immunol. (2010) 125:946–9.e6. 10.1016/j.jaci.2009.12.983 20227752 PMC3412519

[22] Magerus-Chatinet A Stolzenberg MC Loffredo MS Neven B Schaffner C Ducrot N. FAS-L, IL-10, and double-negative CD4- CD8- TCR alpha/beta+ T cells are reliable markers of autoimmune lymphoproliferative syndrome (ALPS) associated with FAS loss of function. Blood. (2009) 113:3027–30. 10.1182/blood-2008-09-179630 19176318

[23] Redman JM Hill EM AlDeghaither D Weiner LM. Mechanisms of action of therapeutic antibodies for cancer. Mol Immunol. (2015) 67(2 Pt A):28–45. 10.1016/j.molimm.2015.04.002 25911943 PMC4529810

[24] Crotty S. T follicular helper cell differentiation, function, and roles in disease. Immunity. (2014) 41:529–42. 10.1016/j.immuni.2014.10.004 25367570 PMC4223692

[25] Futatsugi-Yumikura S Matsushita K Fukuoka A Takahashi S Yamamoto N Yonehara S. Pathogenic Th2-type follicular helper T cells contribute to the development of lupus in Fas-deficient mice. Int Immunol. (2014) 26:221–31. 10.1093/intimm/dxt070 24343821

[26] Besnard C Levy E Aladjidi N Stolzenberg MC Magerus-Chatinet A Alibeu O. Pediatric-onset Evans syndrome: Heterogeneous presentation and high frequency of monogenic disorders including LRBA and CTLA4 mutations. Clin Immunol. (2018) 188:52–7. 10.1016/j.clim.2017.12.009 29330115

[27] Takagi M Hoshino A Yoshida K Ueno H Imai K Piao J. Genetic heterogeneity of uncharacterized childhood autoimmune diseases with lymphoproliferation. Pediatr Blood Cancer. (2018) 65:e26831. 10.1002/pbc.26831 28960754

[28] Rao VK. Approaches to managing autoimmune cytopenias in novel immunological disorders with genetic underpinnings like autoimmune lymphoproliferative syndrome. Front Pediatr. (2015) 3:65. 10.3389/fped.2015.00065 26258116 PMC4508836

[29] Seidel MG. Autoimmune and other cytopenias in primary immunodeficiencies: pathomechanisms, novel differential diagnoses, and treatment. Blood. (2014) 124:2337–44. 10.1182/blood-2014-06-583260 25163701 PMC4192747

[30] Preite S Cannons JL Radtke AJ Vujkovic-Cvijin I Gomez-Rodriguez J Volpi S. Hyperactivated PI3Kdelta promotes self and commensal reactivity at the expense of optimal humoral immunity. Nat Immunol. (2018) 19:986–1000. 10.1038/s41590-018-0182-3 30127432 PMC6140795

[31] Alroqi FJ Charbonnier LM Baris S Kiykim A Chou J Platt CD. Exaggerated follicular helper T-cell responses in patients with LRBA deficiency caused by failure of CTLA4-mediated regulation. J Allergy Clin Immunol. (2018) 141:1050–9.e10. 10.1016/j.jaci.2017.05.022 28601686 PMC5743769

