# Corrigendum: Paradoxical CD4 Lymphopenia in Autoimmune Lymphoproliferative Syndrome (ALPS)

**DOI:** 10.3389/fimmu.2019.01552

**Published:** 2019-07-04

**Authors:** Andrea Lisco, Chun-Shu Wong, Susan Price, Peiying Ye, Julie Niemela, Megan Anderson, Elizabeth Richards, Maura Manion, Harry Mystakelis, Morgan Similuk, Bernice Lo, Jennifer Stoddard, Sergio Rosenzweig, Christophe Vanpouille, Adam Rupert, Irina Maric, Ainhoa Perez-Diez, David Parenti, Peter D. Burbelo, V. Koneti Rao, Irini Sereti

**Affiliations:** ^1^Laboratory of Immunoregulation, National Institute of Allergy and Infectious Diseases (NIAID), National Institutes of Health (NIH), Bethesda, MD, United States; ^2^Laboratory of Clinical Immunology and Microbiology, National Institute of Allergy and Infectious Diseases, National Institutes of Health, Bethesda, MD, United States; ^3^Immunology Service, Department of Laboratory Medicine, National Institutes of Health, Bethesda, MD, United States; ^4^Clinical Genomics Program, National Institute of Allergy and Infectious Diseases, National Institutes of Health, Bethesda, MD, United States; ^5^Laboratory of Immunology, National Institute of Allergy and Infectious Diseases, National Institutes of Health, Bethesda, MD, United States; ^6^Program in Physical Biology, Eunice Kennedy-Shriver National Institute of Child Health and Human Development, National Institutes of Health, Bethesda, MD, United States; ^7^AIDS Monitoring Laboratory, Leidos Biomedical Research, Frederick, MD, United States; ^8^Hematology Service, Department of Laboratory Medicine, National Institutes of Health, Bethesda, MD, United States; ^9^George Washington University Medical Center, Washington, DC, United States; ^10^Dental Clinical Research Core, National Institute of Dental and Craniofacial Research, National Institutes of Health, Bethesda, MD, United States

**Keywords:** CD4 lymphopenia, follicular T helper cells, ALPS-FAS, autoimmune cytopenia, apoptosis

“Peter D. Burbelo” was not included as an author in the published article. The corrected Author Contributions Statement appears below.

“AL, IS, and VR designed research and wrote the paper. AL, C-SW, PY, JN, ER, HM, MS, BL, JS, SR, CV, AR, IM, AP-D, and PB performed research and analyzed data. AL, SP, MA, MM, DP, VR, and IS provided clinical care to enrolled patients”.

The authors apologize for this error and state that this does not change the scientific conclusions of the article in any way. The original article has been updated.

