# Supplementary material for: Paradoxical CD4 Lymphopenia in Autoimmune Lymphoproliferative Syndrome (ALPS)
Source: Front Immunol. 2019 May 29;10:1193. doi: 10.3389/fimmu.2019.01193 (PMC6549489; doi:10.3389/fimmu.2019.01193)
Supplement: Supplementary file 1 [file Table_1.DOCX]

**Supplemental Table 1:**

**Correlation of CD4 and CD8 percentages and absolute numbers with DNT**

| **Comparison** | **Pearson r (95% confidence interval)** | **p** |
| --- | --- | --- |
| **CD4** |  |  |
| **ALPS: Absolute CD4 and DNT counts** | **0.71 (0.40 ⎯ 0.87)** | **0.0003** |
| ALPS CD4 lymphopenic: Absolute CD4 and DNT counts | 0.11 (-0.69 ⎯ 0.79) | ns |
| **ALPS CD4 non-lymphopenic: Absolute CD4 and DNT counts** | **0.76 (0.42 ⎯ 0.91)** | **0.0008** |
|  |  |  |
| **ALPS: Percentages CD4 and DNT** | **-0.67 (-0.85 ⎯ -0.33)** | **0.0008** |
| ALPS CD4 lymphopenic: Percentages CD4 and DNT | -0.47 (-0.90 ⎯ 0.43) | ns |
| **ALPS CD4 non-lymphopenic: Percentages CD4 and DNT** | **-0.74 (-0.91 ⎯ 0.35)** | **0.0021** |
|  |  |  |
| **CD8** |  |  |
| **ALPS: Absolute CD8 and DNT counts** | **0.86 (0.68 ⎯ 0.94)** | **<0.0001** |
| **ALPS CD4 lymphopenic: Absolute CD8 and DNT counts** | **0.83 (0.28 ⎯ 0.97)** | **0.01** |
| **ALPS CD4 non-lymphopenic: Absolute CD8 and DNT counts** | **0.86 (0.61 ⎯ 0.95)** | **0.01** |
|  |  |  |
| ALPS: Percentages CD8 and DNT | -0.04 (-0.46 ⎯ 0.39) | ns |
| ALPS CD4 lymphopenic: Percentages CD8 and DNT | -0.16 (-0.81 ⎯ 0.66) | ns |
| ALPS CD4 non-lymphopenic: Percentages CD8 and DNT | -0.10 (-0.60 ⎯ 0.45) | ns |
